# Pathobiochemistry of Aging and Neurodegeneration: Deregulation of NAD+ Metabolism in Brain Cells

**DOI:** 10.3390/biom14121556

**Published:** 2024-12-06

**Authors:** Nataliya A. Kolotyeva, Alexander A. Groshkov, Nataliya A. Rozanova, Arseniy K. Berdnikov, Svetlana V. Novikova, Yulia K. Komleva, Alla B. Salmina, Sergey N. Illarioshkin, Mikhail A. Piradov

**Affiliations:** Brain Science Institute, Research Center of Neurology, 125367 Moscow, Russia

**Keywords:** nicotinamide adenine dinucleotide, NAD+, metabolism, brain, aging, neurodegeneration

## Abstract

NAD+ plays a pivotal role in energy metabolism and adaptation to external stimuli and stressful conditions. A significant reduction in intracellular NAD+ levels is associated with aging and contributes to the development of chronic cardiovascular, neurodegenerative, and metabolic diseases. It is of particular importance to maintain optimal levels of NAD+ in cells with high energy consumption, particularly in the brain. Maintaining the tissue level of NAD+ with pharmacological tools has the potential to slow down the aging process, to prevent the development of age-related diseases. This review covers key aspects of NAD+ metabolism in terms of brain metabolic plasticity, including NAD+ biosynthesis and degradation in different types of brain cells, as well as its contribution to the development of neurodegeneration and aging, and highlights up-to-date approaches to modulate NAD+ levels in brain cells.

## 1. Introduction

Nicotinamide adenine dinucleotide (NAD+) and its reduced form NADH are essential metabolites with a well-known role in various biological mechanisms, including ATP production, maintenance of genome stability, mitochondrial homeostasis, cell survival, inflammation, and regulation of circadian rhythm. Special attention has been paid to the action of NAD+ and NADH in brain cells because of the high dependence of the central nervous system on the availability of ATP for brain plasticity. NAD+ and its metabolites exert significant effects on synaptic plasticity, cell death and neurogenesis, neoangiogenesis, rearrangement of intercellular communication and intracellular signaling pathways, adaptive response of brain cells to various environmental stimuli, and progression of neurodegenerative disorders, including Alzheimer’s, Parkinson’s, and Huntington’s diseases and amyotrophic lateral sclerosis [1,2].

NAD+ serves as a substrate for a number of proteins, including poly(ADP-ribose)polymerases (PARPs), histone deacetylases–sirtuins (SIRTs), NAD+-glycohydrolases/ADP-ribosyl cyclases (CD38 and CD157), and sterile alpha and TIR motif-containing proteins (SARMs). Using NAD+ as a substrate for their catalytic activity, these proteins act as metabolic sensors within the cell and provide tight interconnection of various subcellular compartments with different metabolic needs. There is a direct link between the redox status of a cell and the control of signal transduction and cell behavior [3,4]. Within the brain, such mechanisms form the basis of brain plasticity; therefore, modulation of NAD+ levels in the brain might be effective for the treatment of conditions associated with aberrant plasticity (e.g., neurodegeneration and aging). Considering that the brain is the most energy-consuming organ in mammals due to its synaptic activity [5], it is not surprising that brain NAD+ levels and the NAD+/NADH ratio are positively associated with ATP production and energy expenditure [6].

Recently, neuroplasticity, which is one of the most important characteristics of the brain’s functional activity and adaptation in the changing environment, has been supplemented with the phenomenon of brain metabolic plasticity [7]. All brain cells—neuronal, glial, endothelial, stem, and progenitor cells—have their own specific metabolic profile and predominant energy-producing mechanisms. For instance, neurons greatly depend on mitochondrial oxidative phosphorylation to support effective synaptic transmission, whereas astrocytes prefer to use glycolysis for generating lactate, which is further used by neighboring neurons or endothelial cells [8]. Total intracellular NAD+ levels and the activity of NAD+-synthesizing enzyme NAMPT control differentiation of neuronal cells and proliferation of stem and progenitor cells in neurogenic niches [9,10,11], whereas NAD+ activity in hypothalamic astrocytes might result in the induction of inflammation [12]. Depletion of NAD+ levels is coupled to the progression of neuronal death [13] and oligodendrocyte loss [14], dysfunction of brain endothelial cells, and blood–brain barrier breakdown [15], but limits astrocyte inflammatory activity [16]. Therefore, understanding specific features of NAD+ metabolism in a particular type of brain cell might explain some contradictory findings on the efficacy of NAD+ supplementation for the prevention of brain aging and neurodegeneration [17].

Several excellent reviews have been recently published on NAD+ metabolism in the brain in (patho)physiological conditions, as well as in aging [1,18,19], and some original studies have confirmed the idea of NAD+ supplementation for better brain functioning [20,21,22]. However, this area of research is one of the most dynamically developing and truly multidisciplinary. Therefore, here we provide an overview of NAD+ metabolism in terms of metabolic plasticity of the brain, in general, and in various types of brain cells, in particular, and focus on recent findings in this area of research.

## 2. NAD+ and NADH Content in Mammalian Cells

The intracellular NAD+/NADH ratio has a significant impact on the regulation of redox reactions. The balance of these two forms is essential for efficient energy metabolism and is regulated by lactate dehydrogenase activity, malate-aspartate, and glyceraldehyde-3-phosphate shuttles [23,24]. The reduced and phosphorylated forms of NAD+ (NADP+ and NADPH) are capable of mutual conversion yet do not affect NAD+ levels. Furthermore, the conversion of NAD+ to NADP+/NADPH is crucial for antioxidant protective mechanisms, maintaining calcium balance in the cell, and thereby affecting cell survival or cell death [25,26,27]. The assessment of this conversion can be easily performed with genetically encoded biosensors available for the assessment of the NAD+/NADH ratio within the cytosolic and mitochondrial compartments [28,29].

Recent studies suggest that a decrease in intracellular NAD+ levels and a deregulated balance of NAD+, NADH, NADP, and NADPH are associated with the progression of aging, including age-associated neurodegeneration and progressive cognitive decline [1,6,30,31]. This might happen either due to imbalanced production and consumption of these nucleotides, or their excessive metabolism and transformation, e.g., in oxidative stress or in overactivation of NADases. Thus, deciphering new aspects of NAD+ metabolism in tissues has become increasingly important due to the potential therapeutic implications of enzymes whose activity depends on the availability of NAD+ in cells.

In mammalian tissues, extracellular NAD+ levels are within the range of 100–500 nM, while intracellular NAD+ content is in the range of 100–120 μM in the nucleus and 50–100 μM in the cytosol. Total cellular NAD+ levels are 200–500 μM. [32,33,34,35]. It is necessary to maintain higher levels of NAD+ in metabolically active cells, such as neurons and cardiomyocytes [36]. The intracellular levels of NAD+ exhibit variability according to a number of factors, including the specific cellular compartment, cell type, cell condition, and growth conditions [34].

The intra- and extracellular levels of NAD+ are closely interrelated, with a complex network of interactions involving the transport of precursors, intermediates, and NAD+ itself across cellular membranes [37]. It is established that NAD+ is unable to cross the lipid bilayer of the cell membrane. Instead, it enters the cell via specific transporters, specifically the connexin 43 (Cx43) channels [38]. A specific transporter protein, MCART1/SLC25A51, is responsible for the delivery of NAD+ to mitochondria. The inhibition of MCART1 gene expression has been observed to result in a notable reduction in tricarboxylic acid cycle activity, mitochondrial respiration, and mitochondrial levels of NAD+ and NADH [39,40].

Extracellular NAD+ also plays a significant role in signaling pathways by interacting with various subtypes of purinergic P2 receptors, including P2Y11, which results in the opening of calcium channels and activation of the cAMP/extracellular Ca^2+^ signaling cascade. This ultimately causes increased cell proliferation and migration [41,42,43]. Furthermore, extracellular NAD+ can interact with postsynaptic P2Y1 receptors, thereby serving as a neurotransmitter or a gliotransmitter [44,45].

Thus, serving as a substrate of NAD+-converting enzymes, NAD+ acquires the characteristics of a pleiotropic signaling molecule. NAD+-dependent enzymes exert their regulatory effects on a multitude of cellular events through ADP-ribosylation and deacetylation of target proteins. These regulatory effects encompass gene transcription, DNA repair, chromatin stability, the cell cycle, differentiation, circadian rhythm regulation, cellular adaptation to stress signals, and immune response. The levels and distribution of NAD+ within the cell can fluctuate in response to a variety of pathological and physiological stimuli. A disruption in the balance of NAD+ is linked to a wide range of pathological conditions affecting multiple organs, including the brain and the nervous system. A decline in NAD+ levels is a defining feature of the aging process and is associated with a range of age-related disorders.

## 3. Basic Mechanisms of NAD+ Metabolism in Brain Cells: Focus on NAD+ Biosynthesis

In mammalian cells, NAD+ is a universal coenzyme and a high-energy compound that is used in electron and proton transfer reactions in mitochondria. NAD+ plays a role in a number of metabolic processes, including glycolysis, the tricarboxylic acid cycle, the synthesis of fatty acids, sterols, and cholesterol, and nucleotide biosynthesis. NAD+ is a direct component of redox reactions. It thus serves as a signaling molecule and a marker of energy availability [46,47]. Furthermore, studies have identified the presence of NAD+ 5′-terminal RNA caps (5′ NAD-RNA) and a NAD+ cap decapping (deNADding) enzyme Xrn1 that modulates RNA stability and mRNA translation [3,48]. Thus, NAD+ bioavailability may control the expression pattern of cells in numerous (patho)physiological conditions.

NAD+ can be produced de novo from tryptophan along with the kynurenine pathway, as well as from precursors such as nicotinamide mononucleotide (NMN) and nicotinamide riboside (NR). Tryptophan may cross the blood–brain barrier and enters the brain cell by the activity of SLC7A5 and SLC36A4 transporters [49,50]. Quinolinate phosphoribosyltransferase (QPRT) represents a pivotal enzyme in the synthesis of NAD+ through the de novo pathway, which is the longest of the NAD+ synthesis pathways. [51]. The de novo pathway contributes only a minor fraction to the total NAD+ pool; however, its importance is highlighted in deficiencies of nicotinic acid and tryptophan where neurological deficits are often registered [52].

The second pathway is the Preiss–Handler pathway where nicotinic acid is used as a precursor of NAD+ and enters the cell by means of SLC5A8 and SLC22A3 transporters. The synthesis occurs in three steps via the intermediate nicotinic acid adenine dinucleotide. Nicotinic acid phosphoribosyltransferase (NAPRT) is the limiting enzyme in the Preiss–Handler pathway [53]. Among the critical enzymes involved in biosynthesis are the nicotinamide mononucleotide adenylyltransferases (NMNATs) NMNAT1, NMNAT2, and NMNAT3. Modulation of these enzymes regulates the cellular levels of NAD. It was proposed that the subcellular distribution of NMNATs may dictate their organelle-specific functions [54,55,56,57].

The salvage pathway from nicotinamide represents the primary pathway of nicotinamide adenine dinucleotide biosynthesis in the majority of mammalian cells. The pivotal regulatory enzyme in NAD+ biosynthesis is nicotinamide phosphorybosyltransferase (NAMPT), which is also known as visfatin and pre-B-cell colony enhancing factor (PBEF). It exists in both intracellular and extracellular forms (iNAMPT and eNAMPT) [58,59]. The intracellular form of iNAMPT takes part in controlling NAD+ levels in cells [60]. For instance, GAPDH and NAMPT form a stable complex essential for the nuclear translocation of NAMPT, thereby supporting the stress-induced NMN/NAD+ salvage pathway [47].

A number of studies have demonstrated that the extracellular form of the enzyme eNAMPT is released from most of the cells, particularly in dysmetabolic or proinflammatory conditions [60,61,62,63]. The eNAMPT protein exists in two distinct structural and functional forms: monomeric and dimeric. Dimerization of NAMPT is a necessary step in the biosynthesis of NAD+. Dimeric phosphoribosyltransferase is present in small extracellular vesicles (exosomes) that can be taken up by recipient cells, thereby increasing the actual NAD+ levels. eNAMPT is less frequently present in the monomeric form and exerts proinflammatory activity [64]. Thus, eNAMPT acts as a danger-associated molecular pattern (DAMP) whose release from affected cells results in the activation of neighboring cells in the tissue. It was demonstrated that eNAMPT in cooperation with interleukin-7 and stem cell factor (SCF) facilitates the formation of pre-B cell colonies [65]. eNAMPT regulates the expression of approximately 50 distinct inflammatory genes in peripheral blood mononuclear cells, stimulating the release of inflammatory mediators, inducing the production of monocyte chemoattractant protein 1 (MCP-1) [66], and affecting the expression of matrix metalloproteinases (MMPs) [67]. In particular, eNAMPT takes part in the activation of nuclear factor NF-κB, mitogen-activated protein kinase (MAPK), and phosphatidylinositol-3 kinase [68]. Ligation of Toll-like receptor 4 (TLR4) by eNAMPT activates NF-κB transcription factor in target cells, which is responsible for the propagation of proinflammatory or pro-aging signaling [69]. By mediating vascular remodeling, eNAMPT activates vascular endothelial growth factor (VEGF) and fibroblast growth factor 2 (FGF-2) [67,70]. Also, eNAMPT functions as a natural antagonist of chemokine receptor type 5 (CCR5) [71]. It has been shown that eNAMPT is more catalytically active than iNAMPT, and adipocyte-released eNAMPT effectively increases NAD+ levels in tissues with low iNAMPT levels, e.g., in pancreatic beta cells and brain neurons [72].

Nicotinamide riboside kinase 1 (NRK1) plays a role in regulating the metabolism of nicotinamide mononucleotide and nicotinamide riboside in cells [73,74]. The extracellular conversion of nicotinamide mononucleotide to nicotinamide riboside by CD73, which is located on the luminal surface of endothelial cells, represents a significant vasoprotective mechanism for maintaining intracellular NAD+ [75].

NAD synthetase 1 is a cytosolic enzyme that catalyzes the final step of NAD+ biosynthesis from tryptophan and nicotinic acid. The enzyme is encoded by the NADSYN1 gene, which is required for the synthesis of NAD+ during the process of embryogenesis [76,77,78].

Table 1 presents a comprehensive overview of the key enzymes involved in NAD+ biosynthesis, including their tissue specificity, functional role, cellular localization, and potential clinical significance.

## 4. Basic Mechanisms of NAD+ Metabolism in Brain Cells: Focus on NAD+ Consumption

The functional role of NAD+ has now expanded beyond its traditional role as a vital coenzyme in energy metabolism. It can now also be viewed as a substrate for a range of proteins, particularly NAD+-converting enzymes, including sirtuin deacetylases (SIRT), poly(ADP-ribose)polymerase (PARP), ADP-ribosyl cyclase/cyclic ADP-ribose hydrolase (CD38/CD157), and NADase SARM1 (Table 2).

Sirtuins (silent information regulator proteins, SIRT 1-7) are NAD+-dependent class III histone deacetylases that play a pivotal role in maintaining and regulating cellular homeostasis. They modulate proteins involved in DNA repair, the cell cycle, aging, neuroprotection, and other processes [117,118]. Of the sirtuins, SIRT1 is the most studied sirtuin in terms of its function, activity, and regulation of various cellular processes. The deacetylation of histones (H1-Lys26, H3-Lys9, and Lys14), non-histone proteins, transcription factors, and cofactors (p53 protein, PPAR-γ, PGC-1α, FOXO3a, FOXO1, and FOXO4) by SIRT1 has been demonstrated to be a crucial process in enhancing mitochondrial metabolism and dynamics, and cell resistance to oxidative stress. SIRT1 regulates cell proliferation, differentiation, survival, inflammation, and apoptosis by modulating the nuclear factor NF-κB and the basic leucine zip (bZIP) family proteins [119,120,121,122,123]. Further evidence indicates that SIRT1 is a pivotal regulator of circadian rhythm-associated metabolism [124,125]. The transcription coactivator PGC-1α has been shown to stimulate mitochondrial biogenesis and catabolism of fat and cholesterol, while also regulating gluconeogenesis and glycolysis. As a result of these actions, mitochondrial mass and functionality are increased, leading to a net increase in energy expenditure [21,126,127]. It was demonstrated that NMNAT3 has the capacity to enhance SIRT3 activity by elevating NAD+ levels, which serves to optimize mitochondrial functionality and rescue the cells from the deleterious effects of oxidative stress [105]. Numerous data confirm that SIRT2 plays a role in mitosis, maintenance of chromatin structure, centrosome integrity, and control of the cell cycle [128,129]. Furthermore, sirtuins affect neurogenesis within neurogenic niches; cyclin-dependent kinases (Cdks) regulate SIRT2 function in the regulation of adhesion and migration of postmitotic hippocampal neurons [130]. SIRT1-dependent autophagy was observed to promote epidermal cell migration and differentiation [131,132].

Poly(ADP-ribose)polymerases (PARPs) constitute a family of proteins comprising 17 enzymes, with poly(ADP-ribose)polymerase 1 and 2 (PARP1 and PARP2) representing the predominant activity within a cell. Several findings indicate that PARP1 and PARP2 activation occurs in response to DNA damage. The prominent role of PARP1 in DNA damage has been shown to induce nucleolar-nucleoplasmic shuttling of the genome maintenance factors WRN and XRCC1 in the cells in a toxicant and protein-specific way [133]. A synergism between PARP1 and phosphorylated Erk, which mediates the expression of the immediate early gene (IEG), has recently been reported in cerebral neurons. This synergism underlies synaptic plasticity and long-term memory acquisition during learning [134]. Therefore, PARPs play a dual role in the cell being able to induce either cell death or DNA repair.

Progression of cell death that occurs in the presence of prolonged DNA damage and excessive PARP1 activation is referred to as “PARthanatos”. This is based on the translocation of free poly-ADP-ribose into the cytosol, where it binds to mitochondrial receptors to stimulate the release of apoptosis-inducing factor 1 (AIF1). AIF1 enters the nucleus and causes subsequent DNA fragmentation [135]. The protease-independent mechanism via HtrA2/Omi is responsible for the regulation of parthanatos [136]. The enzyme PARP1 has an essential role in the regulation of parthanatos, and is a primary source of poly-ADP-ribose in the recognition and repair of single-stranded DNA breaks [135].

Genome integrity is regulated by the bioavailability of NAD+ and the coordinated but independent roles of PARP1, PARP2, SIRT6, DNA ligase II, DNA polymerase, and XRCC1, which are active in DNA repair [137,138,139]. PARP1 is capable of regulating the activity of the DNA polymerase during the process of DNA repair. However, in the absence of NAD+, PARP1 completely inhibits DNA polymerase activity. Upon reaching the physiological level of NAD+, DNA polymerase can resume its repair activity, indicating that the metabolic state of the cell and the integrity of the respiratory chain are cooperative regulatory factors [140].

CD38 and CD157 are multifunctional enzymes that utilize NAD+ as a substrate to generate second messengers, including cyclic ADP-ribose, which plays a role in calcium mobilization, cell cycle control, and insulin signaling. CD38 and its homologue CD157 (BST-1), also known as bone marrow stromal cell antigen-1 (BST-1), were initially characterized as plasma membrane antigens of thymocytes and T-lymphocytes [141,142]. These ectoenzymes possess both ADP-ribosyl cyclase and cyclic ADP-ribose hydrolase activities, and they have been identified in non-lymphoid tissues, including the muscles, liver, and brain [143,144]. CD38 and CD157 fulfil two distinct roles as receptors and ectoenzymes.

The enzymatic extracellular and intracellular functions related to CD38- or CD157-mediated signaling have been extensively characterized [143,145]. Briefly, hydrolysis of NAD+ by CD38 or CD157 results in the generation of molecules with Ca^2+^-mobilizing activity—cyclic ADP-ribose (cADPR) and nicotinic acid adenine dinucleotide 2′-phosphate (NAADP). Elevation of their levels inside the cells leads to Ca^2+^ release from the intracellular store due to interaction with ryanodine receptors (RyRs), so-called hematological and neurological expressed 1-like protein (HN1L)/Jupiter microtubule associated homolog 2 (JPT2) (HN1L/JPT2), respectively, and activation of numerous Ca^2+^-dependent signaling pathways [146]. For instance, in neuronal cells, production of cADPR is required for oxytocin release from hypothalamic and pituitary cells [147], and propagation of signals from activated muscarinic cholinergic and adrenergic receptors [148]. In astrocyes, cADPR controls cell migration [149], progression of reactive gliosis [150], and release of synaptogenic protein SPARCL1 needed for effective synaptogenesis in the postnatal brain [151]. CD38+ or CD157+ microglial cells are present at the loci of neuroinflammation, being activated by various DAMPs and PAMPs; beta-amyloid induces CD38 expression in microglia in the aging brain [152], and a higher prevalence of CD38- and CD157-expressing microglia was found in olfactory bulbs of animals with the experimental model of Alzheimer’s disease [153].

It is widely accepted now that CD38, CD157, and receptor for glycation end products (RAGE) serve as regulators of oxytocin content in the brain and blood [154,155]. A reduction in CD38 expression has been linked to the emergence of autistic traits [22,156,157]. In glial cells, such as astrocytes and microglia, CD38/CD157 plays a role in the regulation of glia-mediated control of neuronal synapse formation and the establishment of social memory [151]. Additionally, it is involved in the activation and polarization of cells during inflammation [158,159], as well as in their migration [160,161,162]. Additionally, CD157 has the capacity to proliferate and is engaged in the processes of angiogenesis and regeneration [144,163]. Transcription factors involved in maintaining the properties of resident vascular endothelial stem cells have been identified [164]. Furthermore, the neuroprotective effect of transplanted CD157-positive endothelial cells has been registered [165].

Moreover, the ectoenzyme CD38 may play a role in the extracellular production of adenosine, being in the functional coupling with CD73 and CD203a [166], but whether such mechanism is operating in the brain remains to be evaluated.

Sterile alpha and toll/interleukin receptor (TIR) motif–containing protein 1 (SARM1) is a metabolic sensor that is activated by NAD+, thereby initiating a program of local destruction in axons. SARM1 activity results in a reduction in NAD+ levels in brain cells through catalyzing the hydrolysis of NAD+ to form nicotinamide, ADP-ribose, and cyclic ADP-ribose [167,168,169]. NAD+ modulates calcium signaling through SARM1 and CD38 in tumor progression, inflammation, and neurodegeneration [170].

The SARM1-Toll/interleukin-1 domain exhibits intrinsic activity for NAD+ cleavage, which contributes to the pathological degeneration of axons [167,171]. Consequently, cADPR serves as an indicator of NADase activity of SARM1 in healthy, injured, and degenerating axons [172].

Two forms of nicotinamide adenine dinucleotide kinase (NADK and MNADK) are encoded within the human genome. These enzymes are also known as NADK2 and are responsible for the phosphorylation of NAD+. MNADK has been demonstrated to serve as a pivotal regulator of acetyl transfer in key metabolic regulators of transcription enzymes; thus, its deficiency impedes the oxidation of fats, resulting in a decline in energy production [173].

**Table 2:** The main NAD+-converting enzymes.

Enzyme	Function	Tissue Expression and Localization in Cell	Clinical Significance
Poly(ADP-ribose)polymerase-1 (PARP1) [174]	The active form of PARP facilitates the transfer of ADP-ribose subunits from NAD+ to protein acceptors, as well as to itself (auto-poly-ADP-ribosylation), resulting in the formation of poly-ADP-ribose chains.	All tissues Neurons [175] Microglial cells [176] Astrocytes [177] Nucleus, cytosol	Neuroinflammation, neurodegeneration [178,179,180], stroke [181], retinal detachment [182], cancer [183]
Poly(ADP-ribose)polymerase-2 (PARP2) [174]			
Sirtuin 1 (SIRT1)	The process of histone deacetylation has been observed to exert a specific influence on the activity of certain transcription factors.	All tissues Neurons [184] Microglial cells, astrocytes [185] Nucleus [117]	Longevity, obesity, diabetes, cancer, cardiovascular and neurodegenerative diseases [119,186,187] ischemic injury [188,189], memory and learning [190,191]
Sirtuin 2 (SIRT2)	Deacetylation of non-histone proteins, mitosis	All tissues Oligodendrocytes, Schwann cells [192] Cytosol [128,193]	Longevity, [186], neuroinflammation [194] neurodegenerative diseases [184,195], synucleinopathies [184].
Sirtuin 3 (SIRT3)	Deacetylation of histones, affect specific transcription factors, regulation of oxidative stress	All tissues Neurons [196] Glial cells [197] Mitochondria [117]	Longevity, metabolic diseases [198,199] ischemic injury [200,201]
Sirtuin 4 (SIRT4)			Metabolic [202], oncological [203] diseases, fibrosis [204]
Sirtuin 5 (SIRT5)			
Sirtuin 6 (SIRT6)		All tissues Neurons [205] Nucleus [117]	Longevity, metabolic diseases [206], fibrosis [207]
Sirtuin 7 (SIRT7)			
CD38	The degradation of NAD+ to form cyclic ADP-ribose and other molecules with calcium mobilizing activities.	All tissues Neurons, astrocytes, microglia, endothelial cells [143] Ectoenzyme, cytosol [143,208]	Cancer, neurodegenerative, metabolic, cardio-vascular, lung, kidney diseases, inflammation [209,210]
CD157			Cancer, neurodegenerative diseases [211]
Sterile alpha and toll/interleukin receptor (TIR) motif–containing protein 1 SARM1	The degradation of NAD+ results in the formation of nicotinamide, ADP-ribose and cyclic ADP-ribose.	Brain, liver, immune cells [167,168]. Neurons [212] Astrocytes [213] Mitochondria [214]	Axonal degeneration [167,215] Neurological diseases [169]
NAD+ kinase	The phosphorylation of NAD+ results in the formation of cytosolic and mitochondrial NADPH.	All tissues Brain [216] Mitochondria	Cancer, neurodegenerative, metabolic, cardio-vascular diseases [173,216,217]

**Figure 1:**
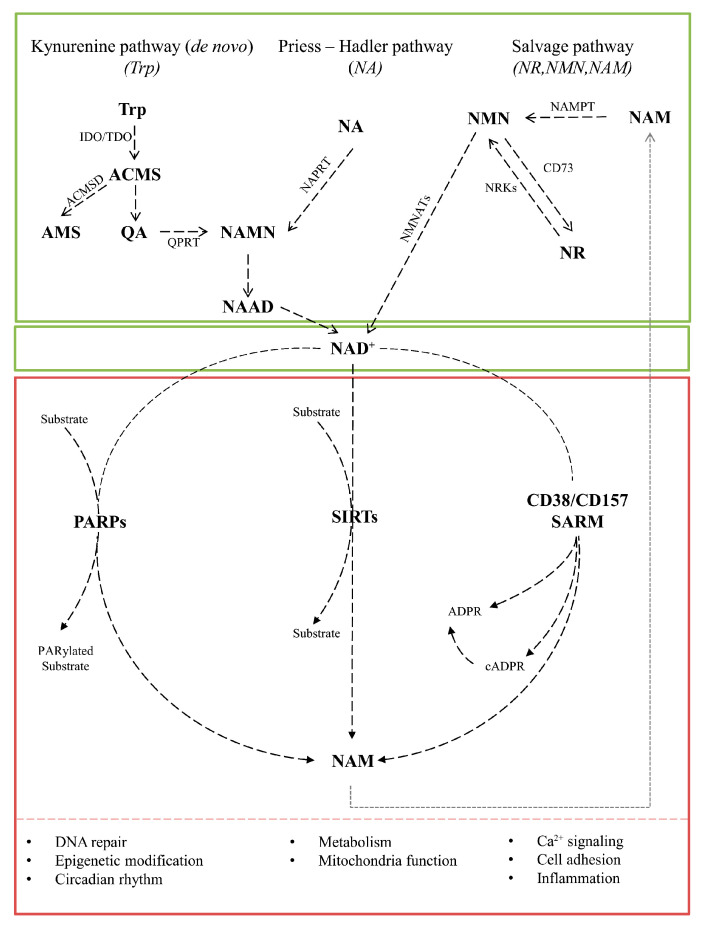
The principal metabolic pathways involved in the synthesis and degradation of NAD+ in cells. The synthesis of NAD+ primarily occurs through three main pathways: the de novo pathway, which involves tryptophan; the Preiss–Handler pathway, where nicotinic acid is used as a precursor of NAD+; and the salvage pathway, which utilizes nicotinamide. The degradation of NAD+ involves hydrolysis and consumption in various enzymatic reactions that play essential roles in cellular metabolism and signaling. The balance between synthesis and degradation is critical for maintaining cellular NAD+ levels, which are vital for energy metabolism and other physiological functions. ACMS—2-amino-3-carboxymuconate semialdehyde; ACMSD—a-amino-b-carboxymuconate-e-semialdehyde decarboxylase; ADPR—ADP ribose; AMS—a-aminomuconate-e-semialdeyde; cADPR—cyclic ADP ribose; CD157—cluster of differentiation 157; CD38—cluster of differentiation 38; IDO—indoleamine 2,3-dioxygenase; NA—nicotinamide; NaAD—nicotinic acid adenine dinucleotide; NAD+—nicotinamide adenine dinucleotide NAM—nicotinamide; NAMN—nicotinic acid mononucleotide; NAMPT—nicotinamide phosphoribosyltransferase; NAPRT—nicotinic acid phosphoribosyltransferase; NMN—nicotinamide mononucleotide; NMNAT1/2/3—Nicotinamide mononucleotide adenylyltransferase type 1/2/3; NR—nicotinamide riboside; NRK—nicotinamide ribonucleoside kinase; PARPs—poly(ADP-ribose) polymerase; QA—quinolinic acid; QPRT—quinolinate phosphoribosyltransferase; SARMs—selective androgen receptor modulators; SIRTs—sirtuins; TDO—tryptophan 2,3-dioxygenase; Try—Tryptophan.

The availability of NAD+ as a substrate determines whether competitive interactions exist between NAD+-degrading enzymes [16,218,219]. In particular, sirtuins are activated in response to nutrient or energy deficiency, prompting cellular adaptation and enhanced metabolic efficiency. Therefore, the activities of SIRT, PARP, CD38/CD157, and SARM1 are mutually competitive with respect to cellular NAD+ uptake. Consequently, overactivation of one enzyme may result in the impairment of the activity of other NAD+-dependent enzymes [105,169,218,220,221,222,223,224]. The principal pathways of NAD+ synthesis and degradation within cells are illustrated in Figure 1.

## 5. The Role of NAD+ in Brain Pathology and Aging

**Figure 2:**
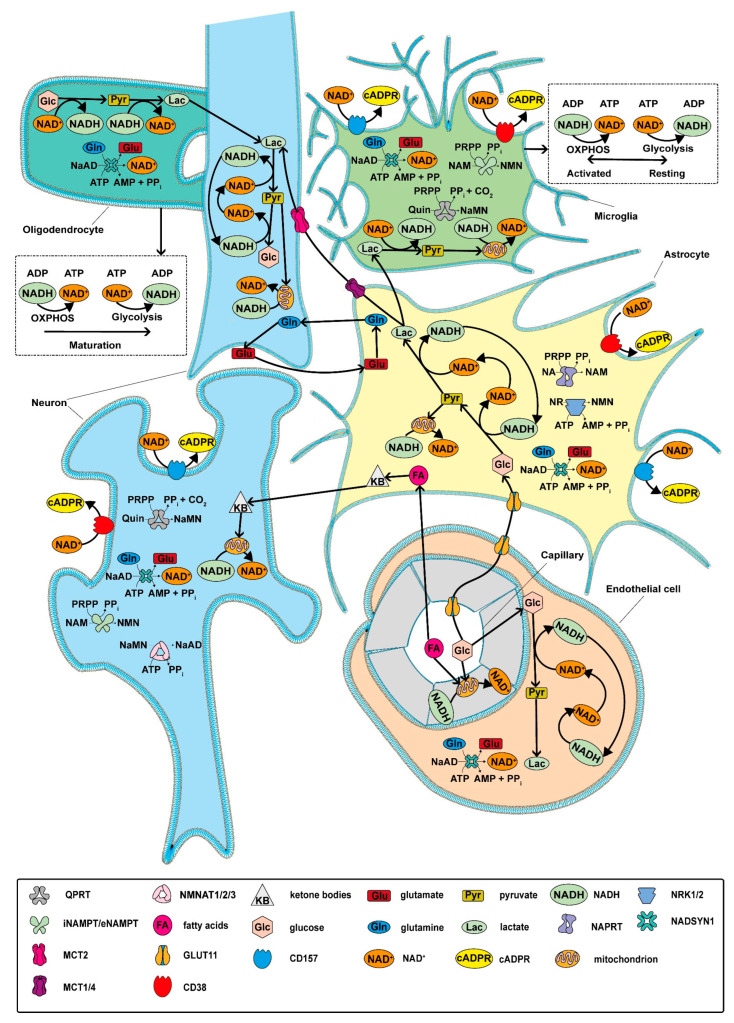
Key aspects of NAD+ metabolism within the neurovascular unit. NAD+ metabolism is of vital importance for the optimal functioning of the neurovascular unit (NVU), exerting a significant influence on both neuronal health and vascular integrity. NAD+ is a vital component of the cellular respiration and ATP production processes in neurons and glial cells, thereby supporting their energy demands. NAD+ plays a role in protecting neurons from oxidative stress and excitotoxicity, enhancing cell survival through the activation of sirtuins and the promotion of DNA repair mechanisms. NAD+ exerts influence over inflammatory responses within the NVU by modulating the activity of immune cells, such as microglia, which can impact neurovascular integrity. In the blood–brain barrier (BBB), NAD+ is involved in maintaining endothelial cell function, regulating permeability, and supporting angiogenesis. Furthermore, NAD+ acts as a substrate for enzymes like PARPs and sirtuins, which are involved in signaling pathways that regulate cell survival, differentiation, and stress responses. ADP—adenosine diphosphate; AMP—adenosine monophosphate; ATP—adenosine triphosphate; cADPR—cyclic ADP ribose; CD157—cluster of differentiation 157; eNAMPT—intracellular nicotinamide phosphoribosyltransferase; FA—fatty acids; Glc—glucose; Gln—glutamine; Glu—glutamate; GLUT1—glucose transporter type I; iNAMPT—intracellular nicotinamide phosphoribosyltransferase; KB—ketone bodies; Lac—lactate; MCT1/2/4—Monocarboxylate transporter type 1/2/4; NA—nicotinamide; NaAD—nicotinic acid adenine dinucleotide; NAD+—nicotinamide adenine dinucleotide oxidized; NADH—nicotinamide adenine dinucleotide reduced; NADSYN1—NAD synthetase type 1; NaMN—nicotinic acid mononucleotide; NMN—nicotinamide mononucleotide; NMNAT1/2/3—Nicotinamide mononucleotide adenylyltransferase type 1/2/3; NR—nicotinamide riboside; PPi—bisphosphate; PRPP—phosphoribosyl pyrophosphate; Pyr—pyruvate; Quin—quinolinic acid.

NAD+ levels and metabolism are critical for the function of several cell types, including neurons, glial cells, and endothelial cells. Neurons are highly dependent on NAD+ for energy production; NAD+ is involved in ATP production through oxidative phosphorylation in mitochondria and affects the activity of sirtuins, which regulate cell survival, stress response, and synaptic plasticity. Glial cells, including astrocytes and microglia, use NAD+ for a variety of purposes: they support neuronal metabolism, maintain the blood–brain barrier (BBB), and support neuroinflammation [225]. Endothelial cell functioning also depends on NAD+ levels to maintain vascular health. NAD+ is involved in the regulation of endothelial nitric oxide synthase (eNOS), an important enzyme in maintaining vascular tone and blood flow. NAD+ availability regulates vascular permeability and angiogenesis, which plays an important role in tissue repair. Reduced levels of NAD+ in endothelial cells lead to endothelial dysfunction and BBB breakdown [15,226]. The metabolism of NAD+ in cells within the neurovascular unit is shown in Figure 2.

A growing body of evidence indicates that aging and a wide spectrum of neurodegenerative disorders, including Alzheimer’s disease, Parkinson’s disease, Huntington’s disease, and amyotrophic lateral sclerosis, are associated with significant metabolic alterations in the brain. These alterations include NAD+ depletion, aberrant cellular bioenergetics, mitochondrial dysfunction, and genomic damage [1]. It has been demonstrated that NAD+, and related metabolites, play a pivotal role in controlling the ability of neurons to adapt to various stress factors and to protect themselves [227]. A non-invasive in vivo NMR-based assay revealed a decline in NAD+, NAD+/NADH, and total NADH levels in the brains of healthy volunteers with advancing age [228]. Furthermore, the energy deficit that arises during the presymptomatic phase of a neurodegenerative disease may be intensified by impaired NAD+ homeostasis, elevated energy expenditure with neuronal network hyperactivity, and glial overactivation [229].

Reduced NAD+ levels are associated with alterations in mitochondrial function and an increase in the production of reactive oxygen species, accompanied by a decline in oxidative metabolism [36]. Subsequently, there is an increase in mitochondrial dysfunction, a switch from oxidative phosphorylation to glycolysis, an accumulation of hypoxia-inducible factor 1 alpha (HIF-1α), and a decrease in the activity of respiratory complexes in mitochondria. These changes have been linked to the development of metabolic syndrome, obesity, type 2 diabetes, tumor progression, and neurodegeneration [36,230].

NAD+ induces the transcription of stress and metabolic genes through the modulation of circadian rhythms [231]. Aging is associated with decreased NAMPT expression, impaired regulation of circadian rhythm via the CLOCK/BMAL machinery, high PARP activity associated with DNA damage, development of inflammatory or metabolic stress, an increase in CD38 activity and the subsequent decrease in NAD+ levels, and impaired mitochondrial function via the SIRT3-dependent mechanism [232,233]. Furthermore, CD38 plays a regulatory role in the generation of hydrogen peroxide in the aging brain. This is achieved through the modulation of pyruvate dehydrogenase and alpha-ketoglutarate dehydrogenase, which are located within mitochondria and are responsible for H_2_O_2_ leakage sites [234]. Thus, CD38 NADase plays a pivotal role in the age-related reductions in NAD+ levels in the brain. Regulation of NAD+ synthesis in the hypothalamus by eNAMPT has been demonstrated to play a pivotal role in the control of circadian rhythms. This system may be significantly impaired in obesity, because obesity can disrupt circadian fluctuations in the plasma eNAMPT−hypothalamic NAD+-FOXO1 axis [89,235]. It can therefore be seen that circadian rhythms are generated by the internal mechanisms of a cell that regulate a number of various physiological and metabolic events. The stability of circadian rhythms is weakened during in aging. Also, SIRTs and mTOR exert their effects on mechanisms that control the aging process [236,237]. Thus, it is tempting to speculate that lowered NAD+ availability in hypothalamic neurons may underly the aging-associated changes in the regulation of circadian rhythms.

Some studies [84,85] highlight an association of the pathogenesis of epilepsy, Huntington’s disease, and stroke with aberrant activity of quinolinate phosphoribosyl transferase (QPRT). This enzyme plays a significant role in neuroprotective mechanisms against quinolinic acid-induced excitotoxicity. The rise in quinolinic acid is linked to an increase in the activity of enzymes involved in the kynurenine pathway, coupled with a reduction in the activity of the enzyme phosphoribosyl pyrophosphate synthetase. The utilization of phosphoribosyl pyrophosphate for de novo synthesis results in a reduction in its availability for NAD+ synthesis. The kynurenine pathway plays a role in modulating neuronal functions. This is due to its involvement in the metabolism of the two major neurotransmitters, glutamate and acetylcholine, the regulation of NMDA receptors’ activity, and the generation of reactive oxygen species [83,238]. In Parkinson’s disease (PD), there is generally an imbalance in the levels of kynurenine (KYN), kynurenic acid (KYNA), and quinolinic acid (QUIN). Studies have shown that KYN and QUIN levels are elevated in PD patients, but KYNA levels are decreased in the cerebrospinal fluid. These changes may lead to increased neurotoxicity due to the action of QUIN, which is known to induce excitotoxicity and contribute to neuronal damage [239]. QUIN interacts with NMDA receptors, resulting in neuronal damage due to calcium influx. KYNA may act as a neuroprotectant by modulating local immune reactions. Thus, an imbalance in these metabolites leads to neuroinflammation and neurodegeneration in PD [240]. Moreover, in PD, LRRK2 G2019S mutation in dopaminergic neurons reduces NAD+ levels, which is associated with decreased sirtuin activity and acetylation of key substrates [220].

Neuronal and astroglial cells exposed to cytotoxic levels of glutamate and quinolinic acid exhibited an increase in intracellular oxidative stress and PARP activity. Since PARP1 is involved in a number of other processes, including differentiation, cell proliferation [241], regulation of DNA repair [242], cholinergic and glutamatergic signal transduction and metabolism [243], and memory consolidation [244], deregulation of PARP-mediated NAD+ metabolism results in apoptosis and autophagy of brain cells. Modulation of PARP1 activity has been shown to regulate region-specific astroglial responses, which is crucial for maintaining synaptic activity [177,245]. The parthanatos cell death pathway mediates early brain damage and is closely linked to stroke [181]. It was observed to play a role in photoreceptor death in retinal detachment [182]. Consequently, the mutations and dysfunction of PARP activity have been linked to a range of pathologies, including malignancies, neurodegenerative diseases (such as ataxia-telangiectasia and Cockayne syndrome, as well as Xeroderma pigmentosum group A), brain injuries, inflammatory diseases, and metabolic disorders [178,179,180,246].

Inhibition of PARP-1 has been found to improve symptoms and reduce neurodegeneration in a number of AD models, including in Drosophila; therefore, treatment with Olaparib—a PARP-1 inhibitor—has shown neuroprotective effects through reduction in β-amyloid aggregates and enhancement in neuronal function [247] Moreover, nicotinamide, already a known PARP-1 inhibitor, may present a potential therapeutic strategy in early AD, as it enhances NAD+ levels and diminishes neuroinflammation [248]. Evidence also suggests that inhibition of PARP-1 could decrease oxidative stress and mitochondrial dysfunction associated with neurodegeneration [246,249]. In this regard, targeting PARP-1 is one of the most promising approaches for developing therapies that could delay the progression of AD and other neurodegenerative diseases [246].

The NAD+-glycohydrolase (NADase) activity of CD38 should be considered as a metabolic and redox sensor in mammalian cells because of its ability to control the intracellular NAD+ levels and bioavailability of NAD+ for the action of other NAD+-dependent enzymes, e.g., PARPs or sirtuins [250]. It was found that CD38 upregulation in tumor cells produces significant NAD+ depletion, impairing mitochondrial fitness and provoking oxidative stress [251]. In aging cells, higher expression of CD38 results in mitochondrial SIRT3-dependent NAD+ depletion and mitochondrial dysfunction [233]. Since mitochondrial sirtuin 3 is widely expressed in the brain, where it protects neurons from neurotoxic stimuli and prevents microglia senescence [252], it is tempting to speculate CD38-Sirt3 machinery might be responsible for the aging-associated mitochondrial dysfunction in brain aging.

Very recent research suggests that NADase activity of CD38 in ovarian cells is the key determinant of ovarian aging associated with the establishment of the SASP phenotype, even if no specific changes in the expression of other NAD+ consumption genes were detected [253]. In myocardial aged cells, CD38 downregulates Sirt6 expression and promotes cell senescence [254]. It is probable that a similar mechanism might be seen in the brain, since, in neurons, fully functional Sirt6 prevents cell senescence, and supports synaptic plasticity and cell survival, but affects inflammatory response being expressed in microglia [252].

SIRT1 supports neurites growth, axonal development, and dendrite branching. These mechanisms are known to affect consolidation of long-term memory and learning [190,191]. The administration of NAD+ was observed to improve cognitive function and mitigate neuroinflammation in models of chronic cerebral hypoperfusion in rats. This was achieved by protecting mitochondria and reducing the production of reactive oxygen species via the SIRT1/PGC-1α pathway [21,255]. A number of studies have reported a correlation between reduced SIRT1 expression and the development of Alzheimer’s disease and depression. Serum SIRT1 is able to cross the blood–brain barrier and is considered a promising potential serum biomarker for the early detection of Alzheimer’s disease [187,256]. SIRT2 has been shown to prevent α-synuclein-mediated toxicity in PD by deacetylating lysine 6 and 10 of α-synuclein, thereby preventing its aggregation and toxicity [184]. A deficiency in SIRT1 in the mouse brain has been observed to alter the distribution of site-specific phospho-tau in synaptosomes, thereby mediating a synaptic tauopathy in Alzheimer’s disease [257].

SIRT1 is involved in the metabolism of the amyloid and tau proteins in AD pathophysiology. It can regulate the activity of various enzymes critical for APP metabolism, thereby reducing beta-amyloid production. For instance, SIRT1 regulates autophagy and tau deacetylation, thereby preventing accumulation of beta-amyloid and hyperphosphorylated tau protein in the tissue [258]. SIRT1 possesses anti-inflammatory activities, which also play an important role in maintaining proper brain health by inhibiting NF-κB-mediated expression of genes encoded for pro-inflammatory cytokines in neuroinflammation [259].

The regulatory effects exerted by SIRT1 and SIRT3 on the permeability of the human blood–brain barrier have been demonstrated in response to hypoxia [260,261]. NMNAT1 has been demonstrated to support the integrity of the blood–brain barrier following cerebral ischemia via the NAD+/SIRT1 signaling pathway [188,189].

SIRT1 restores mitochondrial function via SIRT3 activation, triggers autophagy through regulation of the AMPK-mTOR pathway in ischemic brain injury [200,201], and modulates DNA replication in senescent cells [262]. Phosphorylated SIRT1 inhibits the initiation of replication, thereby maintaining genomic stability [262]. The depletion of SIRT3 and SIRT7 has been demonstrated to result in the disruption of nuclear integrity, loss of heterochromatin, and accelerated senescence of human stem cells [198,206,263].

The expression and activity of NAMPT have been demonstrated to be elevated in a number of pathological conditions, including ischemic brain injury [264], inflammation [265], and metabolic diseases [266]. The catalytic activity of NMNAT plays a crucial role in the inhibition of neurodegenerative processes both in vitro and in vivo. It has been demonstrated that NMNAT isoforms may serve as chaperones [103]. The overexpression of NMNAT has been observed to reduce the levels of hyperphosphorylated tau oligomers, thereby preventing behavioral and morphological abnormalities associated with tauopathy [267]. A notable reduction in NMNAT3 protein levels has been identified in individuals diagnosed with Parkinson’s disease, and this has been linked to an increase in monomeric α-synuclein. This indicates that modifications in the NAD+ biosynthesis pathway are implicated in α-synuclein-mediated synaptopathy [107]. In Huntington’s disease, NMNAT restores neuronal integrity by neutralizing the progressive toxicity caused by mutant huntingtin aggregates [268]. This protective function is independent of NAD+ synthesis activity, indicating the potential involvement of direct protein–protein interactions [269].

NMNAT2 plays a crucial role in maintaining glycolysis through maintaining NAD+ homeostasis [2,270]; thus, it is reasonable that a reduction in the levels of NMNAT2 has a detrimental effect on the development and survival of axons [99]. The NMNAT2 maintains vesicular glycolysis, thereby enabling rapid axonal transport. A reduction in SARM1 activity protects against deficits in axonal transport and slows the progression of axonal degeneration [2,271].

Certain mutations in *PARP* reduce mitochondrial toxicity caused by toxic protein aggregation in AD and HD [272,273]. In addition, mutations in *PARP1* genes have been associated with changes in the susceptibility and severity of Alzheimer’s disease; therefore, targeting NAD+ metabolism and PARP activity may be a therapeutic option in the treatment of neurodegenerative diseases.

Reductive stress in brain cells is a consequence of alterations in the balance of oxidized and reduced forms of pyridine nucleotides (lower levels of NAD+ and higher levels of NADH). This condition may be caused by a number of factors, including suppressed activity of NAMPT, altered glycolysis, and inhibited OXPHOS. As a result, PARP and SIRTs function less efficiently, and irreparable DNA damage accumulates, leading to replicative stress [274,275]. Reductive stress impacts mitochondrial function and results in increased production of mitochondrial reactive oxygen species (ROS), which in turn leads to protein misfolding, lipid peroxidation, and DNA damage [272,276,277]. ROS-induced DNA damage has been demonstrated to trigger mechanisms of cellular senescence. Furthermore, ROS initiate oxidative damage of the tight junction machinery in brain endothelial cells, activation of matrix metalloproteinases, and BBB breakdown. As a result, neuroinflammation occurs, thereby supporting release of DAMPs from damaged cells and progression of aging [273,278,279]. The accelerated aging in neurodegeneration is manifested with impairment of brain plasticity [277,280].

Systemic and cerebral insulin resistance supports progression of neurodegeneration. It has been demonstrated that mice on a high-fat diet exhibit a NAMPT-dependent reduction in NAD+ synthesis. Knockout of NAMPT results in the development of insulin resistance, whereas NMN administration has been demonstrated to increase muscle NAD+ turnover and insulin sensitivity in a group of overweight or obese postmenopausal women with prediabetes [281,282]. Furthermore, CD38 deficiency has been demonstrated to enhance glucose tolerance [225,283]. In the brain, local insulin resistance is always associated with the progression of neurodegeneration due to altered signal transduction from insulin receptors widely expressed in neuronal, glial, and endothelial cells [284,285].

**Figure 3:**
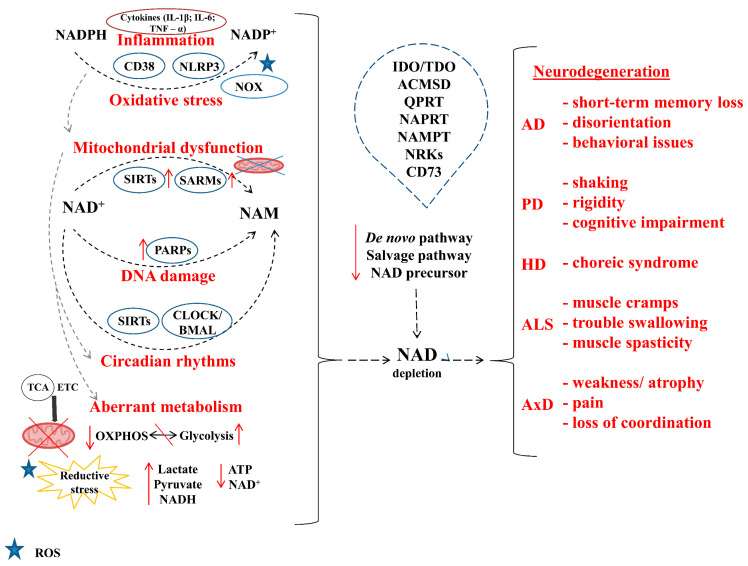
The altered metabolism of NAD+ in neurodegeneration and aging. The altered metabolism of NAD+ in neurodegeneration and aging is characterized by a decline in NAD+ levels, which has a deleterious effect on cellular functions. In neurodegenerative disorders, reduced availability of NAD+ impairs energy production, DNA repair, and antioxidant defenses, thereby increasing neuronal vulnerability and cell death. Furthermore, this decline affects sirtuins, which regulate stress responses and inflammation, thereby exacerbating neuroinflammation and contributing to disease progression. Additionally, the process of aging serves to further exacerbate these issues. The reduction in NAD+ that occurs naturally with age disrupts the metabolic pathways that are essential for maintaining neuronal and vascular health. This ultimately results in the promotion of cognitive decline and neurodegenerative processes. ACMSD—a-amino-b-carboxymuconate-e-semialdehyde decarboxylase; AD—Alzheimer disease; ALS—amyotrophic lateral sclerosis; AxD—Alexander disease; ATP—adenosine triphosphate; BMAL—brain and muscle arnt-like protein 1; CD 37—cluster of differentiation 37; CLOCK—circadian locomotor output cycles kaput; ETC—electron transport chain; HD—Huntington disease; IDO—indoleamine 2,3-dioxygenase; NAM—nicotinamide; NAMPT—nicotinamide phosphoribosyltransferase; NLRP3—NOD-like receptor protein 3 inflammasome; NOX—NADPH oxidases; NRK—nicotinamide ribonucleoside kinase; PD—Parkinson disease; OXPOHS—oxidative phosphorylation; SIRT—silent information regulator protein; SARM—Sterile alpha and toll/interleukin receptor (TIR) motif–containing protein 1; QPRT—quinolinate phosphoribosyltransferase; TDO—tryptophan 2,3-dioxygenase.

A summary of NAD+-dependent mechanisms in brain aging and neurodegeneration is shown in Figure 3. All the above data support the opportunity to apply NAD+-based therapies to neurodegeneration and brain aging [279].

## 6. Current and Prospective Pharmacological Strategies: Targeting the Brain’s Metabolic Plasticity via NAD+ Producing and Consuming Reactions

The existing literature provides evidence that agents that increase NAD+ levels in brain neurons demonstrate neuroprotective effects, and are helpful in reducing neurodegeneration and demyelination in disease models of Parkinson’s disease, Alzheimer’s disease, and multiple sclerosis [1,19,286]. Some studies have indicated the potential for lifestyle and dietary factors such as fasting, caloric restriction, and physical activity to regulate NAD+ levels; [287,288], however, these strategies may not be applicable to all patient groups. The following pharmacological approaches for the modulation of NAD+ levels are widely discussed: (1) inhibition of NAD+ consumption by targeting NAD+-converting enzymes, including PARP, CD38, and CD157 [289,290,291,292]; (2) stimulation of NAD+ biosynthesis through the action of enzymes that generate NAD+ [286,293,294]; (3) use of NAD+ precursors, such as nicotinamide riboside, nicotinamide mononucleotide, nicotinic acid, nicotinamide, and tryptophan [293]; (4) application of NADH dehydrogenase modulators, such as β-lapachone [295]; and (5) use of natural products in boosting NAD+ (apigenin, fisetin, embelin) [296,297,298].

### 6.1. Inhibition of NAD+ Consumption

A number of studies have shown that the inhibition of NAD+-degrading enzymes is an effective method of increasing NAD+ levels. A specific inhibitor of CD38, 78c, has been demonstrated to decelerate the age-related diminution of NAD+ through the activation of factors associated with longevity and health, including sirtuins, AMP-activated protein kinase, PARPs, the inhibition of mTOR-S6K, ERK, the attenuation of DNA damage, and cellular senescence. It reduces insulin resistance and improves glucose tolerance, and supports muscle and cardiac function and exercise capacity in physiological and accelerated aging [289,290,291].

The inhibition of CD38 activity and the addition of nicotinamide riboside has been demonstrated to affect NF-κB signaling pathways in microglia. This may represent a potential strategy for the suppression of neuroinflammation and subsequent neurodegeneration [299]. An anti-CD38 antibody, TNB-738, increases intracellular levels of NAD+ and the activity of sirtuins. This drug has broad therapeutic potential and offers several advantages, including a long half-life, specificity, and safety [221].

In the context of therapy in brain ischemia, neurodegenerative diseases, and aging, PARP inhibitors represent a promising avenue for further study, both in vitro and in vivo. Inhibition of PARP has been demonstrated to enhance mitochondrial metabolism and autophagy through the activation of SIRT1 [292,300]. This has been shown to be an effective method of protecting primary cortical neurons from oxygen–glucose deprivation, whereby mitochondrial NAD+ is preserved by preventing its release into the cytosol [301,302,303]. The study indicated that the administration of a PARP-1 inhibitor (AG14361) via the intraperitoneal route can restore the neurological function, diminish oxidative stress, preserve the integrity of the blood–brain barrier, and attenuate the expression of proteins linked to inflammation and neuronal apoptosis [181].

NAD+ can prevent age-related damage of the blood–brain barrier through the Cx43-PARP1 mechanism [15]. The inhibition of PARP1 by olaparib or nicotinamide mononucleotide restores NAD+ levels in cells, thereby reducing the BBB permeability associated with aging [15]. In a further study, the inhibition of the mitochondrial NAD+ transporter SLC25A51 was demonstrated to facilitate PARP1-dependent DNA repair by elevating nuclear NAD+ levels within the cell [304], thereby suggesting the potential utilization of the SLC25A51 transporter as a target for regulating NAD+ transport [305,306].

### 6.2. Stimulation of NAD+ Biosynthesis

It is possible to enhance the level of NAD+ by modulating the enzymes responsible for its synthesis, and NAMPT represents a promising target. The NAMPT activator, aminopropylcarbazole (P7C3), exhibits good pharmacokinetic properties, high bioavailability, and the capacity to avert neuronal destruction in neurodegenerative disorders [307]. P7C3 derivatives were developed to assess their proneurogenic activity in vivo. It was demonstrated that these compounds elevate the intracellular concentration of NAD+, which in turn triggers metabolic and transcriptional reprogramming with minimal toxicity. This finding paves the way for the development of novel neuroprotective pharmaceutical agents [308,309,310].

The screening of NAMPT activators has identified NAMPT-positive allosteric modulators (N-PAM), which have been shown to increase enzyme activity and NAD+ levels in the cell. Furthermore, improvements in the synthesis process have resulted in the creation of an orally bioavailable N-PAM, which may prove invaluable for future studies and potential clinical applications [310,311].

Initially, NAMPT inhibitors were investigated as NAD+-reducing drugs for the treatment of cancer. Several studies have demonstrated that these compounds possess anti-inflammatory properties and might have some neuroprotective effects [312,313]. A protective effect has been demonstrated in mouse models of spinal cord injury and brain ischemia [264,265]. Furthermore, its administration in aged mice resulted in enhanced cognitive performance and elevated motor activity, which is likely attributable to augmented autophagy and reduced aggregation of cellular proteins in the brain. These findings suggest potential for this treatment in the management of neurodegenerative disorders [312,314].

A novel NAMPT inhibitor that selectively targets NAPRT-deficient EMT-subtype cancer cells and provides relief from chemotherapy-induced peripheral neuropathy is currently being studied. Inhibitor A4276 has been demonstrated to safeguard axons from Wallerian degeneration with greater efficacy by diminishing the ratio of nicotinamide-mononucleotide to NAD+, underscoring its potential as a promising anticancer agent [315,316]. The pharmacological inhibition of SARM1 has been demonstrated to protect axonal structure and function in peripheral neuropathy [171,317]. Furthermore, SARM1 inhibitors may prove beneficial in the treatment of bone pathology in diabetes [318].

### 6.3. Using NAD+ Precursors

An alternative strategy for modulating NAD+ involves the use of NAD+ precursors. These include nicotinamide riboside, nicotinamide mononucleotide, nicotinic acid, nicotinamide, tryptophan, and other compounds. Mitochondrial dysfunction and oxidative stress have been shown to be central to the progression of neurodegeneration [319]. Increased NAD+ levels due to supplementation with nicotinamide riboside (NR) or vitamin B3 improve mitochondrial function in brain cells [320]. NAD+ precursors, including nicotinamide riboside and nicotinamide mononucleotide, are naturally occurring compounds found in a variety of foods, including vegetables, meat, and milk. Additionally, these precursors can be produced by microorganisms in fermented beverages [321]. The systemic administration of nicotinamide mononucleotide has been demonstrated to effectively enhance NAD+ biosynthesis in a range of peripheral tissues [322,323,324]. The intraperitoneal administration of nicotinamide mononucleotide to experimental animals has been demonstrated to increase NAD+ levels in the hippocampus and hypothalamus, thereby improving cognitive function in rats with a model of Alzheimer’s disease [325]. Nicotinamide mononucleotide has been found to prevent neuronal death, improve mitochondrial function, promote energy, and reduce the accumulation of reactive oxygen species [326,327,328]. Furthermore, it has been demonstrated that nicotinamide mononucleotide has positive effects on general metabolism, including insulin secretion and impaired glucose tolerance. This latter effect is of particular importance in the context of the prevention and treatment of type 2 diabetes and other metabolic diseases [329,330,331,332]. It is probable that it might be also helpful for the treatment of local insulin resistance in the brain, which is a key feature of Alzheimer’s-type neurodegeneration [153,285].

Mutations in the genes *KYNU*, *HAAO*, and *NADSYN1* have been identified in patients presenting with congenital malformations, which have been defined as a congenital NAD+ deficiency disorder (CNDD). The aforementioned mutations result in a plethora of severe malformations. A deficiency in the *NADSYN1* gene results in a reduction in the intracellular levels of NAD+, which can be caused by a lack of precursors such as tryptophan and nicotinic acid [76,77,78]. Treatment strategies have been proposed, including the addition of amidated NAD+ precursors (nicotinamide or nicotinamide mononucleotide) to the diets of pregnant women, with continued supplementation required postnatally [78].

The potential deleterious effects of nicotinamide mononucleotide (NMN) supplementation on programmed axon death require further studies. A substantial body of evidence indicates that a reduction in NMNAT2 activity results in elevated nicotinamide mononucleotide levels and the activation of the pivotal pro-degenerative enzyme SARM1, which may subsequently contribute to axonal degeneration. Conversely, nicotinamide mononucleotide and other NAD+ precursors have been the subject of considerable interest with regard to the development of anti-aging therapies [169,333,334,335].

The anti-inflammatory role of nicotinamide mononucleotide in brain and spinal cord injury has been elucidated. It affects signaling pathways associated with inflammation, including IL-17, TNF, TLR, NOD-like receptor, and chemokines, thereby inhibiting the expression of inflammatory factors [336,337]. The ability of nicotinamide riboside to inhibit leukocyte chemotaxis, and its capacity to enhance the immune microenvironment, were observed in mice. These effects were found to promote neuronal survival and facilitate motor function recovery following spinal cord injury [338,339]. Moreover, nicotinamide riboside has been demonstrated to impede the age-related diminution of dopaminergic neurons and motor dysfunction in animal models of PD [340]. Additionally, it has been shown to mitigate neuroinflammation and cellular senescence in a model of Alzheimer’s disease [341]. Therefore, oral administration of a NAD+ precursor has a beneficial effect on the recovery of social behavior in CD157 knockout mice [342]. However, the available evidence on the oral administration of nicotinamide riboside and its effects on metabolism in humans is inconclusive. It was observed that the administration of the compound increased the levels of NAD+ in the skeletal muscle of elderly individuals, while simultaneously reducing the levels of circulating proinflammatory cytokines [343]. In other studies, the administration of nicotinamide riboside in a dietary supplement form has been demonstrated to exert no impact on skeletal muscle mitochondria in obese and insulin-resistant men [344,345]. Further studies using different demographic groups and longer-term treatment are needed to establish the real potential of nicotinamide riboside supplementation [346].

The elevation of NAD+ levels by the application of a NADH dehydrogenase modulator, such as β-lapachone, a metabolite that modulates cellular NAD+ by conversion of NADH to NAD+ via the enzymatic action of NADH: quinone oxidoreductase 1 (NQO1), has been demonstrated to effectively prevent age-related hearing impairment in rodents. This is achieved by reducing the levels of inflammatory mediators and oxidative stress, while simultaneously supporting mitochondrial function and stimulating mitochondrial biogenesis [295,347].

**Figure 4:**
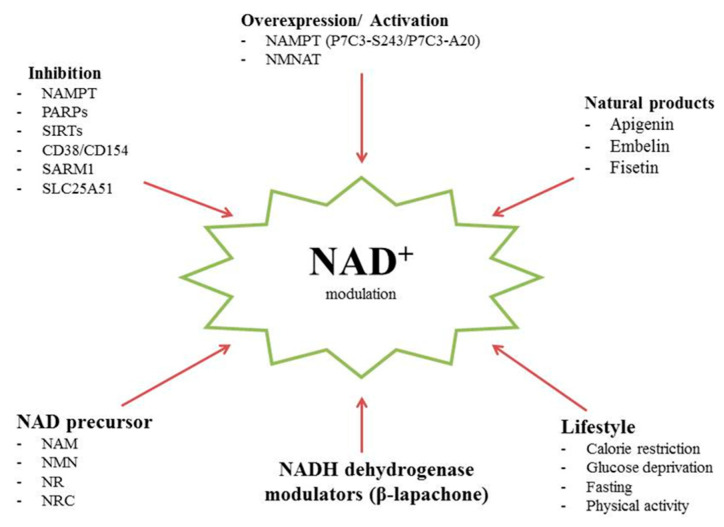
NAD+-based modulation strategies with neuroprotective potential. The administration of NAD+ precursors has been demonstrated to elevate NAD+ levels, thereby enhancing mitochondrial function and cellular energy. The selective targeting of NAD+-converting enzymes such as PARP and CD38 can prevent the excessive consumption of NAD+ during cellular stress, thereby ensuring its continued availability for vital processes. Strategies to enhance the recycling of NAD+ from its metabolites can assist in maintaining cellular NAD+ levels and supporting neuroprotection. These strategies seek to restore cellular homeostasis, reduce neuroinflammation, and improve overall brain health, offering potential therapeutic avenues for age-related cognitive decline and neurodegenerative disorders.

Figure 4 presents a summary of the main NAD+-based modulation strategies.

## 7. Conclusions

NAD+ is a vital metabolite that is fundamental to a number of biological mechanisms, including energy metabolism, genome stability, and cell survival. NAD+ has been demonstrated to play a pivotal role in brain plasticity and the metabolic plasticity of brain cells in general, and their adaptation to stress by functioning as a sensor and regulator of energy availability. The multiplicity of biological effects resulting from NAD+ metabolism serves to illustrate its fundamental role as a signaling molecule [46]. Of particular interest is the assessment of the potential role of NAD+ metabolism proteins in various neurodegenerative diseases known as diseases of advanced aging, as well as in physiological aging. This suggests that NAD+-synthesizing and -converting enzymes could be used as promising therapeutic targets. Therefore, understanding the molecular and cellular mechanisms of NAD+ homeostasis and its role in different types of brain cells will facilitate the development of novel therapies to promote healthy brain functioning and aging. The utilization of compounds that specifically modulate the activity of enzymes engaged in NAD+ metabolism will facilitate the elucidation of the role of NAD+ in (patho)physiology. It will provide a basis for the development of novel drugs for the correction of chronic neurodegeneration and neuroinflammation.

The growing interest in healthy lifestyles and active longevity has led to a surge in interest in the use of NAD+ precursors as dietary supplements. Further studies are required to ascertain the optimal dosage, duration of treatment, potential for adverse effects, and risk groups that may be affected. It is essential to consider the pharmacokinetics and pharmacodynamics of NAD+ modulators, including their bioavailability, metabolism, and tissue distribution. Better understanding of these aspects will facilitate the optimization of their use, thereby preventing any potential adverse effects. This is essential for ensuring the safety of strategies designed to increase NAD+ levels in aged individuals and those with neurological deficits.

## Figures and Tables

**Table 1:** The key enzymes involved in the biosynthesis of NAD+.

Enzyme	Function	Tissue Specificity, Localization in Cell	Clinical Significance
Quinolinate phosphoribosyltransferase (QPRT) [51]	Secondary rate-limiting step in the de novo synthesis of NAD+: conversion of quinolinic acid to nicotinic acid mononucleotide (kynurenine pathway).	Liver, kidney, brain [51,79] Microglial cells [80] Neurons [81] Cytosol [82]	Epilepsy, Huntington’s disease [83], stroke [84], inflammation and infection [85]
Nicotinamide phosphoribosyltransferase intracellular (iNAMPT) [58,59]	It performs a biosynthetic function and participates in the synthesis of nicotinamide mononucleotides in the salvage pathway.	Adipose tissue, muscle, heart, liver, kidney, brain [86] Neurons [87] Microglial cells [88] Cytosol, nucleus, mitochondria [89]	Type 2 diabetes, [90], multiple sclerosis [16], neurodegeneration [91]
Nicotinamide phosphoribosyltransferase extracellular (eNAMPT) [58,59]	It is known to circulate as extracellular vesicles, which have the capacity to undergo endocytosis by recipient cells, thereby increasing NAD+ levels and cytokine production in the salvage pathway.	Adipose tissue, liver, macrophages, cancer cells, heart [92] Neurons, microglial cells [92] Ectoenzyme, cytosol and nucleus [93]	Cardiovascular and metabolic disorders [89,90], inflammation: sarcoidosis [94], colitis [95], lung injury [96], cancer [61,93]
Nicotinamide mononucleotide adenylyltransferase 1 (NMNAT1) [56]	The conversion of nicotinic acid mononucleotide to nicotinic acid adenine dinucleotide occurs in the de novo, Preiss–Handler pathway.	Skeletal muscles, heart, kidneys, liver, pancreas [54,55] Neurons [97] Nucleus [98]	Axonal degeneration [57]
Nicotinamide mononucleotide adenylyltransferase 2 (NMNAT2) [56]		Neurons [99,100] Cytosol, endosomes, synaptic endings, axons [55,101,102]	Axonal degeneration [99,103]
Nicotinamide mononucleotide adenylyltransferase 3 (NMNAT3) [56]		Erythrocytes, lungs, spleen [104,105]. Neurons [106] Mitochondria, cytosol [54,55]	Synucleinopathies [107]
Nicotinic acid phosphoribosyltransferase (NAPRT) [53]	The conversion of nicotinic acid to nicotinic acid mononucleotide in the Preiss-Handler pathway.	Large intestine, heart, kidney, liver Endothelial cells, astrocytes [108] Cytosol and nucleus [109]	Sepsis, septic shock [110]
Nicotinamide riboside kinase 1 (NRK1)	The phosphorylation of nicotinamide riboside results in the formation of nicotinamide mononucleotide in the salvage pathway.	Liver, kidneys, muscles [74,109] Astrocytes, neural progenitor cells (NPCs) [111] pancreatic β-cells [112] Mitochondria	Diabetes [112] Liver steatosis [113]
Nicotinamide riboside kinase 2 (NRK2)		Heart, brain [114], muscles [74] Mitochondria	Cardiovascular and metabolic disorders [114]
NAD synthetase (NADSYN) [76]	The final step of de novo biosynthesis, the Preiss–Handler pathway, involves the amidation of adenine dinucleotide nicotinic acid.	All tissues [1,111] Brain [111] Cytosol	Congenital NAD+ deficiency is associated with the *NADSYN1* gene, the development of organ malformations [78,115,116]
